# Profiling Volatile Constituents of Homemade Preserved Foods Prepared in Early 1950s South Dakota (USA) Using Solid-Phase Microextraction (SPME) with Gas Chromatography–Mass Spectrometry (GC-MS) Determination

**DOI:** 10.3390/molecules24040660

**Published:** 2019-02-13

**Authors:** Lucas J. Leinen, Vaille A. Swenson, Hope L. Juntunen, Scott E. McKay, Samantha M. O’Hanlon, Patrick Videau, Michael O. Gaylor

**Affiliations:** 1 Department of Chemistry, Dakota State University, Madison, SD 57042, USA; ljleinen@pluto.dsu.edu (L.J.L.); hope.juntunen@trojans.dsu.edu (H.L.J.); Scott.McKay@dsu.edu (S.E.M.); 2 Department of Biology, Dakota State University, Madison, SD 57042, USA; Vaille.Swenson@trojans.dsu.edu; 3 School of Psychological Science, Oregon State University, Corvallis, OR 97331, USA; ohanlons@oregonstate.edu; 4 Department of Biology, Southern Oregon University, Ashland, OR 97520, USA

**Keywords:** historical foods, preserves, volatile organic compounds (VOCs), bisphenol-A (BPA)

## Abstract

An essential dimension of food tasting (i.e., flavor) is olfactory stimulation by volatile organic compounds (VOCs) emitted therefrom. Here, we developed a novel analytical method based on solid-phase microextraction (SPME) sampling in argon-filled gas sampling bags with direct gas chromatography–mass spectrometry (GC-MS) determination to profile the volatile constituents of 31 homemade preserves prepared in South Dakota (USA) during the period 1950–1953. Volatile profiles varied considerably, but generally decreased in detected compounds, complexity, and intensity over three successive 2-h SPME sampling periods. Volatile profiles were generally predominated by aldehydes, alcohols, esters, ketones, and organic acids, with terpenoids constituting much of the pickled cucumber volatiles. Bisphenol-A (BPA) was also serendipitously detected and then quantified in 29 samples, at levels ranging from 3.4 to 19.2 μg/kg, within the range of levels known to induce endocrine disruption effects. Absence of BPA in two samples was attributed to their lids lacking plastic liners. As the timing of their preparation coincides with the beginning of BPA incorporation into consumer products, these jars may be some of the first BPA-containing products in the USA. To the best of our knowledge, this is the first effort to characterize BPA in and volatile profiles of rare historical foods with SPME.

## 1. Introduction

Human olfactory sensing of volatile organic compounds (VOCs) released from foodstuffs (i.e., their volatile profile) is an essential component of the perception of flavor [1]. When sealed foods are opened and prepared, VOCs spanning a multitude of functional classes interact synergistically to generate the aromatic and gustatory properties of the food. Given the variety of food types and associated volatile inventories (and the potential complexity thereof), it is difficult to directly relate human perceptions of, for example, “sweet” and “savory” tastes to specific volatile functionalities. However, based on general trends in the literature, the sweet aromas of fruits and vegetables appear to be defined primarily by volatile profiles enriched in, for example, aldehydes, alcohols, esters, ketones, quinones, and terpenes [2,3], while savory aromas, e.g., of meats and cheeses, tend to be defined by volatile profiles more enriched in sulfur- and nitrogen-containing compounds (e.g., thiols, sulfides, and amines) [4,5].

Evidence of food preservation by humans using primitive techniques (e.g., burial storage and sun drying, etc.) dates to 20,000 BC [6], with the first evidence of liquid preservation via pickling dating to ancient Greek and Egyptian cultures [7]. Many contemporary food preservation techniques are now liquid-based, and necessitate boiling and subsequent sealing of metal or glass vessels, and appear to be derived from those developed in more recent times (circa 300 years ago) [8]. These methods—generically referred to as “canning”—involve the immersion of foods in acidic brine and/or sugar solutions with subsequent heating and sealing to prevent microbial degradation. Such techniques have since been practiced and evolved by cultures around the globe, including the early pioneer settlers of the circa mid-19th-century North American frontier, who required simple, effective methods for longer term food storage prior to the advent of in-home refrigeration [9].

It is unlikely that food preservation was also intended as a method of preserving records of past chemical environments, but it occurred to us during conception and design of this study that it may be a fortuitous outcome and worthy of exploration. During the canning process, some chemical constituents originating from that point in time (e.g., via atmospheric deposition or plant-associated soil components, etc.) may be preserved along with the foods, creating a sort of chemical “time capsule” that might yield interesting insights into the nature of those environments [10,11]. To the best of our knowledge, there are no published reports of solid-phase microextraction (SPME) applied to archaeological studies of the volatile constituents of canned or other prepared foodstuffs. However, SPME applications in archaeology appear to be increasing [12,13,14], and reports of other analytical/chemical methods applied to archaeological studies of historical foods and food remains dates back to the late 20th century [10,11,15,16,17,18,19,20]. 

Inspired by the potential to obtain new insights into the food preservation techniques and possibly the environmental conditions of the North American Great Plains region during this time, and to address the lack of SPME studies of historical foods, we sought to profile the volatile inventories of a variety of homemade preserved foods prepared in Moody County, South Dakota (SD; USA) during the period 1950–1953. A secondary aim of the study was to quantify burdens of the toxic plasticizer bisphenol-A (BPA), discovered serendipitously in pursuit of the above primary study aims. To the best of our knowledge, this is the first reported effort to use SPME to profile the volatile constituents of rare preserved historical foods sealed for more than three generations, as well as the first report of a SPME-detectable toxic compound therein. 

## 2. Results and Discussion

### 2.1. SPME Method Development

**Figure 1:**
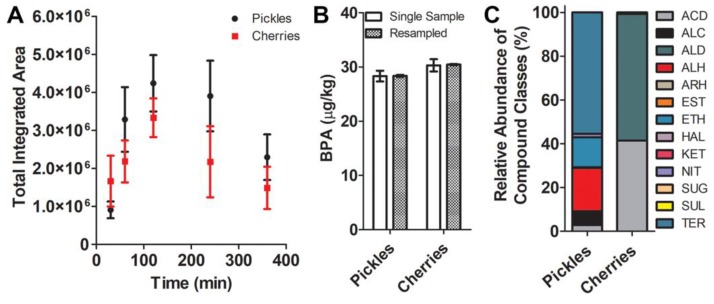
Validation of a solid-phase microextraction (SPME) method to assess the volatile inventories of preserved foods using commercially available dill pickles and maraschino cherries. A time course from 30–360 min of SPME sampling was conducted for both pickles (black circles) and cherries (red squares) in triplicate. Total integrated peak area is presented as the average ± standard deviation (**A**). As the 120 min (2 h) timepoint produced the greatest total integrated peak area for both preserved foods, the reproducibility of the sampling protocol was assessed based on the level of BPA measured following spiking 1000 μg into jars of pickles and cherries (**B**). Five jars each of pickles and cherries were sampled a single time, and three different jars of each food were sampled in three successive replicates. The concentration of BPA was determined by comparison to a standard curve and is presented as the average ± standard deviation for single samplings (clear bars) and average ± standard error of the mean for replicate samplings (dotted bars). The relative abundance of the assessed compound classes (as percent of total) is presented for the pickles and cherries sampled for 2 h with SPME (**C**). Compound classes are defined as follows: ACD = acids; ALC = alcohols; ALD = aldehydes; ALH = aliphatic hydrocarbons; ARH = aromatic hydrocarbons; EST = esters; ETH = ethers; HAL = halogen-containing; KET = ketones; NIT = nitrogen-containing; SUG = sugar alcohols; SUL = sulfur-containing; TER = terpenoids.

Once the initial batch of 1950s preserves was collected, it quickly became evident that there were few studies of, and no published methods to assess, the VOCs from these types of rare historical food samples. To design and validate a method to profile the volatile constituents from these irreplaceable historical preserves, SPME sampling was used to define the optimal sampling time for preserved foods, and to verify that the compounds identified indeed originated from the samples being assessed. Store-bought preserved foods were used to assess the time to equilibrium uptake of VOCs in the AtmosBag because these foods were locally available, are presumed to have highly consistent volatile profiles across individual jars based on the batch preparation of such foods, and could be purchased in sufficient quantities to permit replicate single samplings. Lacking a priori knowledge of the composition of their volatile profiles, savory and sweet preserved foods (dill pickles and maraschino cherries) were selected to encompass a wide range of representative compound functionalities during method development. Store-bought dill pickle and cherry preserves were sealed in the sampling bag along with the SPME device, and sampled in triplicate for 30, 60, 120, 240, and 360 min as described in the Experimental Section (Figure S1). Equilibrium uptake (as measured by maximum total integrated peak area) was achieved by 120 min for both pickles and cherries (Figure 1). This sampling time was used for all subsequent SPME samplings. Sampling efficacy was further validated by assessing uptake of authentic bisphenol-A (BPA) standards spiked into both store-bought matrices and sampled with SPME for 120 min. Reproducibility between samples and within triplicate resampling replicates differed by less than 5% (Figure 1), confirming method efficacy.

The SPME method permitted detection of 20 unique compounds, constituting the Vlasic dill pickle volatile inventory, that conformed to our analytical detection and identification parameters (see Experimental Section). The volatile profile of the Vlasic dill pickles was dominated by acetic acid, ammonium acetate, alcohols, esters, and ketones, and the terpenoids tentatively identified as β-myrcene, α-phellandrene, 4-carene, *p*-cymene, limonene, α-terpineol, and d-carvone (Table S1). The absence of unsaturated aldehydes, e.g., (*E*,*Z*)-2,6-nonadienal and (*E*)-2-nonenal, in the Vlasic volatile profile was surprising, as these compounds have been reported to be essential aroma compounds in pickled cucumber preparations [21]. In contrast to the 20 compounds identified from Vlasic dill pickles, the maraschino cherry volatile inventory was comprised of only 4 compounds, tentatively identified as benzaldehyde, (*Z*)-3-hexenol acetate, ascorbic acid, and benzoic acid (Supplementary Data File 1), which is consistent with known cherry volatile profiles [22,23].

To validate the integrity of the AtmosBag, further assess the efficacy of the SPME method, and assess the extent to which the individual components of a pickled cucumber preparation contributed compounds to the measured volatile inventory, we characterized the VOCs of store-bought cucumbers (sliced into spears and stored in clean Vlasic dill pickle jars to keep system dimensions constant), a lab-prepared artificial pickling brine (used to approximate the Vlasic dill pickle brine), and a lab preparation of the cucumber spears pickled in the artificial brine for 7 days (see Experimental Section). Each of the components and the mixture thereof was analyzed in triplicate with the SPME method. As published volatile profiles for commercial pickle preparations are lacking, this was done to show that the major compounds detected in the Vlasic pickles were consistent with those detected in the individual components, and not sampling artifacts or environmentally-derived compounds, providing additional verification of method efficacy. The cucumber spears alone produced only 2 compounds, tentatively identified as limonene and 3,6-dimethyl-2,3,3a,4,5,7a-hexahydrobenzofuran, while the artificial brine and the cucumber spears pickled therein combined to produce a volatile inventory enriched with most of the major compounds detected in the Vlasic dill pickles with the established method (Table S2). The 20 compounds detected here are consistent with the 21 compounds reported to comprise the volatile inventory of a competing brand of dill pickles [24]. However, only a few compounds detected here were reported in that study (acetic acid, ethyl acetate, α-Terpineol). This is likely due to differences in brine composition and SPME fiber phase used (75 μm Carboxen- polydimethylsiloxane (PDMS) phase). 

Important aroma/flavor volatiles detected in the Vlasic pickles included acetic acid, ammonium acetate, β-myrcene, α-phellandrene, 4-carene, *p*-cymene, limonene, and d-carvone, further confirming the efficacy of our SPME method to capture the major volatile constituents of this commercial dill pickle preparation. Though these compounds appear typical of preserved cucumber preparations, the relatively simpler volatile profile was also a bit surprising, in that previous studies have reported abundances of, for example, longer chain alcohols and unsaturated aldehydes in cucumber volatile emissions [25]. This is presumably due to the different SPME fiber phase and sampling approaches, as well as the different cultivars, used in that study. The presence of limonene may be an artifact of the chitosan coatings commonly applied to fresh cucumbers to extend shelf life [26], or perhaps a metabolic response to stress induced by the physical process of preparing the cucumbers for study [27]. This experiment could not be performed with the maraschino cherries, as it was not possible to obtain store-bought Marasca cherries (or other types used for this recipe), and the complex ingredients listed on the store-bought cherries were not reproducible in the lab. However, the capacity of the SPME method to reproducibly detect major compounds reported to comprise the volatile inventories of Marasca cherry [23] and commercial dill pickle preparations [24] over all sampling times and replicate sampling periods provided compelling support for the validity and efficacy of the method developed here to assess the major compounds of the historical preserves. Additional confirmation of method validity was attained via the BPA spiking experiments (Figure 1). 

### 2.2. SPME Analysis of 1950s Preserves

**Figure 2:**
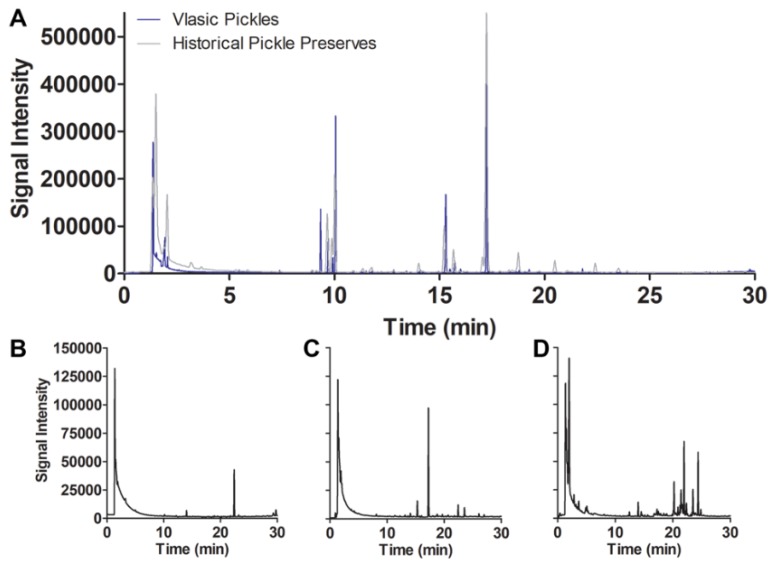
Total ion chromatograms (TICs) derived from 2 h SPME sampling of representative samples. Vlasic dill pickle (blue line) and historical preserve dill pickle type 2 (sample 29; gray line) TICs are overlaid to show similarities and differences in compounds detected and their intensities (**A**). Representative TICs of low (tomato sample 2; **B**), medium (dill pickle sample 30; **C**), and high (sweet pickle sample 31; **D**) complexity samples. The *y*-axis labels and intensity presented in the panel B TIC are the same for panels C and D.

**Figure 3:**
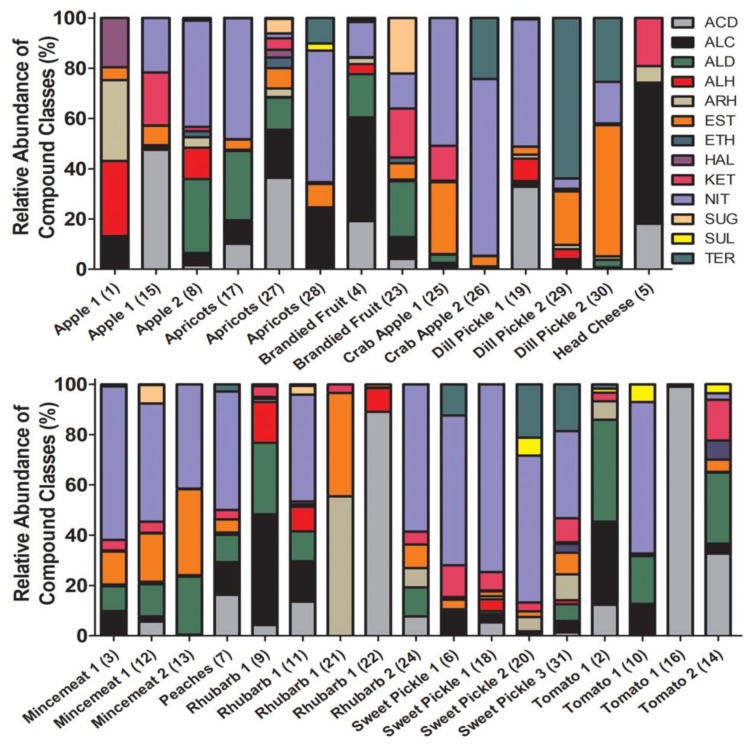
The relative abundance of the assessed compound classes (as percent of total) in the historical preserves. Samples are ordered alphabetically by type, and the sample number is in parenthesis after the sample type. Compound classes presented as colors are defined as follows: ACD = acids; ALC = alcohols; ALD = aldehydes; ALH = aliphatic hydrocarbons; ARH = aromatic hydrocarbons; EST = esters; ETH = ethers; HAL = halogen-containing; KET = ketones; NIT = nitrogen-containing; SUG = sugar alcohols; SUL = sulfur-containing; TER = terpenoids.

After validating the SPME sampling method for jars of commercially preserved foods from the present day, the technique was applied to the historical samples for which it was developed (31 jars of historical preserves; Figure S2). The volatile profiles of the 1950s preserves varied considerably in number of compounds detected, class composition, and intensity over the sample set and over three successive SPME samplings. Total ion chromatograms (TICs) showing the volatile profiles of representative low, medium, and high complexity determined during the first SPME sampling are presented in Figure 2B–D. Numbers of compounds varied from as few as 2 (rhubarb, sample 24) to as many as 67 (sweet pickle, sample 31) detected in the first sampling period, with compound numbers, total integrated peak area, and volatile profile complexity generally decreasing over successive SPME samplings (Figure 3, Figure S3). The first SPME sampling period was of particular interest, because it is presumed to represent the total VOCs from the production time period before exogenous compounds (i.e., gases) were introduced upon repeated samplings. Of the samples, 74% displayed a lower total integrated peak area in the third sampling than in the first, and 26% of these had a higher total integrated peak area in the second sampling than in the first sampling. Interestingly, 22% of the preserves showing decreased integrated peak area in successive samplings produced a higher number of compounds in the third sampling than in the first sampling, which indicates that new compounds were evolved from these samples over successive SPME sampling periods. Compound class composition and diversity was also highly variable, as one might expect from homemade food preserves sealed for three generations (Figure 3, Figure S3). Compound class diversity was lowest in tomato preserves (sample 16) and highest in apricot preserves (sample 27).

TICs of the first SPME sampling of the Vlasic dill pickle volatiles were compared to a 1950s dill pickle volatile profile to assess compound relatedness (an overlay of their TICs is shown in Figure 2A). Similar to the Vlasic pickles, the 1950s dill pickle volatile profile was predominated by acids, alcohols, esters, and ketones, but the latter produced nearly twice the number of compounds, including a larger inventory of aliphatic and aromatic hydrocarbons. However, the 1950s sample produced a comparable inventory of terpenoid compounds (Table S2). This was also one of only two historical preserve samples to produce detectable levels of the compound tentatively identified as apiol (a phenylpropanoid isolated from parsley with known abortive effects), the other being a sweet pickle preserve.

Consistent with their bulk acidic nature (Table 1), the volatile profiles of nearly all the preserves contained organic acids, but acid species predominated in apple, apricots, dill pickle, rhubarb, and tomato preserves. Acetic acid was the dominant acid in the “less sweet” dill pickle, rhubarb, and tomato preserves. The volatile profiles of sweet preserves were generally predominated by esters, followed by aldehydes, consistent with studies of modern sweet foodstuffs [22]. Three of the four rhubarb samples were exceptions to this trend, consistent with our taste test classifications of “slightly sweet” (Table 1). Much of the ester inventory is presumed to arise from esterification reactions involving abundant free fatty acids likely produced and solubilized to higher concentrations during heating of the foods prior to canning [28]. The prevalence of esters followed by aldehydes in dill pickles is generally consistent with those reported for the volatile inventories of modern cucumbers and pickled cucumbers [21,24]. 

Aldehydes are recognized as impactful aroma components in foods, which may derive from Maillard reactions occurring during the heating of foods prior to sealing. Aldehydes may also arise from oxidation of unsaturated fatty acids via peroxidation, which can drive production of, for example, the increased alcohol and hydrocarbon inventories [28] also detected in these samples. In support of this explanation, peroxides were also detected in the aldehyde enriched preserves, but did not meet the established criteria for positive compound identification (described in Experimental Section). Aliphatic hydrocarbons were also detected in nearly all samples, but were prevalent in apples, brandied fruit, dill pickles, and rhubarb, several of which also contained appreciable aldehydes. Alkanes constituted the majority of the aliphatics, which may also be explained in part by degradative reaction cascades originating with Maillard reactions [28]. 

Alcohols are also recognized as important aroma compounds in many food types, but are generally less abundant in food volatiles relative to, for example, aldehydes, esters, and terpenes. Alcohols are also important precursors for aldehyde and ester production, which may account for their lesser abundance in the volatile inventories of foods [28]. In this study, alcohols were prevalent in apricots, brandied fruit, head cheese, rhubarb, and tomato samples. The preponderance of alcohols in head cheese is consistent with the volatile inventories reported for some types of meats [29]. Like alcohols, ketones may be essential aroma compounds in foods, but are generally less abundant relative to, for example, aldehydes, esters, and terpenes [22,30,31]. In this study, ketones were prevalent in apple, brandied fruit, crab apple, head cheese, mincemeat, peaches, rhubarb, sweet pickle, and tomato preserves, consistent with ketone inventories reported for other fruit- and meat-based foodstuffs [29,32].

Terpenoids were detected sporadically in multiple preserves, but were prominent constituents in some dill pickle, sweet pickle, and crab apple preserves (Figure 3, Figure S3, Table S2), consistent with previous reports of volatile profiles of analogous foods [21,24,33]. Terpenoids produce aromas in foods previously described as, for example, “sweet,” “rose-like,” “green,” “fruity,” “citrus,” “piney,” “floral,” “resinous,” “lemon,” and “lemon-like” [34]. These descriptions are generally consistent with our own smell and taste characterization of the preserves (see Supplemental Video V1). Surprisingly, these terpenoids were absent in the other crab apple (sample 25), dill pickle (sample 19), and sweet pickle (sample 18) preserves. It is not clear why such substantial compositional differences would be detected in such seemingly similar preserves, but this may be indicative of quite different preparation methods, or variations in the individual fruits/vegetables harvested from the rural home garden during this time period. 

Nitrogen-containing compounds were prevalent in most of the preserves, but were conspicuously absent from apple (sample 1), head cheese (sample 5), rhubarb (samples 21, 22), and tomato (samples 2, 16, 14) (Figure 3). The most commonly occurring compounds were tentatively identified as hydrazides of acetic acid (detected in apple, apricots, dill pickle, mincemeat, rhubarb, sweet pickle, tomato), butyric acid (detected in apple, mincemeat, rhubarb), and formic acid (detected in mincemeat, rhubarb, sweet pickle), ammonium acetate (detected in dill pickle, mincemeat, sweet pickle), *N*,*N*-dibutylformamide (detected in apricots, brandied fruit, peaches, rhubarb, tomato), (Z)-9-octadecenamide (detected in rhubarb), and hydrazine derivatives (detected in apricots, brandied fruit, crab apples, mincemeat, sweet pickle, tomato). Reports of nitrogen-containing volatiles in preserved foods are limited, but are presumed to be driven by Maillard reactions between amino acids and sugar alcohols during preparation, and other degradative reactions involving amines, amino acids, peptides, and alkaloids thereafter. Sulfur compounds were minor constituents of only a few preserves (detected in apricots, sweet pickle, tomato), and were tentatively identified as sulfurous acid esters and alkyl sulfides, consistent with sulfur compounds reported in sweet, tangy, and savory foods [35]. 

### 2.3. BPA in 1950s Preserves

**Figure 4:**
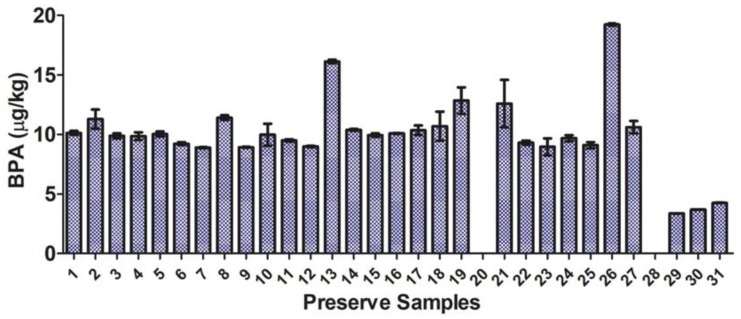
The concentration of bisphenol-A (BPA) in historical preserves is presented as the average ± standard deviation determined from three successive samplings of each historical preserve.

Detection of BPA in all the preserves was surprising, as this compound was just being introduced into consumer products in the early 1950s as an additive in the protective epoxy resin coatings of food containers [36]. These jars may thus represent some of the earliest BPA-containing consumer products in the USA. BPA was detected in the volatile profiles of all preserves (but below the quantitation limit of 1 μg/kg in samples 20 and 28—sweet pickle and apricots), and was detected in all sampling replicates at levels ranging from 3.4 to 19.2 μg/kg (Figure 4). Such low BPA levels in samples 20 and 28 was interesting, and is presumed to be the result of different lids (lacking plastic liners) being used to seal these preserves. These preserves were not sealed using the Ball canning lids used on all other preserves (Figure S2), supporting the idea that the incurred BPA was derived from the plastic liners of the Ball lids. 

These samples notwithstanding, detected BPA levels were in the range of those reported to induce endocrine disruption (e.g., antiandrogenic) effects in biochemical assays [37]. BPA levels were fairly constant over the entire sample set and over the three successive samplings of each preserve (Figure S4), consistent with reported persistence of release pathways from consumer polymers [38] and our data from resampling experiments using BPA spiked into store-bought preserves (Figure 1). Though significant differences in SPME-detected BPA burdens were detected (*p* < 0.05; paired *t*-test), if one considers the absolute mass of BPA sampled by the SPME fiber, detected masses were considerably more uniform over the data set (Figure 4; Figure S4; Table S3). Such uniform volatilization from the preserves over lengthy successive SPME sampling periods provides potentially useful insight into human exposure risks prior to regulatory controls on BPA. Indeed, these may be the first such insights available in the literature. 

Using spiked BPA uptake data, we estimated naturally incurred BPA burdens (presumed to have leached from the protective plastic lid liners during storage) to be of the order of 1000 μg sample^−1^. This estimate is within the range of measured concentrations of BPA leached from consumer bottles lined with epoxy resin-based protective coatings [39]. The absolute mass of spiked BPA sampled by the SPME fiber over the entire sample set was calculated to be 2.5 ± 0.2% of the total spiking mass (1000 μg), which is consistent with BPA’s low vapor pressure and the Henry’s Law constant. It is also in general agreement with uptake efficiencies predicted for the 100 μm PDMS phase possessing an absorptive sampling volume of the order of 10^−9^ L [40] (sampling volume of the AtmosBag is a billion times larger), and those previously reported for structurally analogous aromatic pollutants [41]. 

The data presented indicate that the compound class compositions profiled in these rare historical preserves are in general agreement with those determined in many studies of modern preserved food types. This is particularly interesting given that the historical preserves assessed here were prepared before the widespread use of ultra-pasteurization, UV sterilization, or even the freeze-drying methods used to preserve many commercial foods. The general similarities in class compositions suggests that the method(s) used to prepare these historical preserves and those described elsewhere were not sufficiently different to produce radically distinct SPME-detectable volatile profiles. Alternatively, if modern food preservation methods are vastly different from those used to prepare these preserves during the period 1950–1953, those differences were not manifested in wholly different SPME-detectable volatile profiles. It is also possible that, because the overall class compositional profiles were comparable between modern and historical preserves, the cultivars preserved were not different enough to evolve entirely disparate volatile profiles. Though differences at the level of individual compounds were certainly apparent, the trends in compound classes detected with the SPME method suggests overarching similarities with modern preserved foods.

Detection of BPA in all of these historical foodstuffs provides an unprecedented glimpse into what may have been some of the earliest incidences of human exposure to this toxic compound, originally believed to be tightly sequestered in consumer polymers and thus biologically inaccessible. Finally, to the best of our knowledge, this is the first account of a simple, reproducible, and robust SPME method for profiling volatile inventories and industrial pollutant burdens of rare canned historical foodstuffs. Taken together, these study findings indicate that SPME may be a powerful, but so far underexplored, analytical tool for elucidating the chemical constituents of archaeological samples more generally. 

## 3. Experimental Section

### 3.1. Chemicals, Equipment, and Ingredients

All solvents and reagents were of analytical grade (purity > 99%) and were purchased from Sigma-Aldrich (Saint Louis, MO, USA). The SPME sampler, SPME fibers, and sampling bags (Aldrich® AtmosBag) were also purchased from Sigma-Aldrich (product number Z564427). A 24 gauge, 100 μm polydimethylsiloxane (PDMS) SPME fiber was used to sample all preserves. This phase was selected because it has been shown to be one of the most versatile and rugged fiber phases for sampling a wide range of compound functionalities in demanding environmental sampling applications (e.g., field analyses of challenging environmental matrices in Tedlar bags) [42]. This phase has also proven effective for characterizations of the volatile and semi-volatile profiles of food and beverage matrices [5,43]. Additionally, PDMS is similar to the GC column phase (DB-5) used here, has greater film thickness (permitting increased uptake capacity), is generally more reproducible over larger numbers of samplings, and exhibits greater thermal resilience over numerous/longer high temperature injection times (personal communication, Sigma-Aldrich applications chemists; [44]). PDMS has also been described as a good general phase for profiling volatiles/semi-volatiles when one is uncertain as to which fiber phase is most appropriate for a given analysis and suite of compounds (personal communication, Sigma-Aldrich chemists; [40,42]). Finally, prior to profiling the preserves with this fiber, we conducted preliminary sampling trials of store-bought dill pickle and maraschino cherry preserves with the 100 and 7 μm PDMS and 85 μm polyacrylate fibers (purchased as an assortment kit from Sigma-Aldrich), and found that the 100 μm PDMS fiber sampled the greatest number of compounds and produced the largest total chromatographic area for these sample types. 

Analytical grade (99.999% purity) helium and argon were purchased from A-OX Welding Supply (Sioux Falls, SD, USA). Dill pickles (Vlasic Kosher Dill Spears), maraschino cherries (Best Choice), cucumbers, and brine ingredients (white vinegar (ShurFine), dill seeds (McCormick), and table salt (ShurFine)) used to develop and validate the SPME method were purchased from a local supermarket (Sunshine Foods, Madison, SD, USA). Sample pH was determined using an Accumet Excel XL15 pH meter (Fisher Scientific, Waltham, MA, USA).

### 3.2. Sample Collection, Curation, and Preparation

Following discussions with the Moody County Historical Society (Flandreau, SD, USA) Director and its Board of Trustees, access to a circa 1895 constructed bare-earth basement (near Flandreau, SD) housing the preserved foods was arranged by a local family in August 2016 and again in August 2018. Therein, an estimated 125 glass jars of varying types and sizes containing preserved foods were stacked on hand-made wooden shelves assembled along the north-facing wall (Supplemental Video V2). The homeowner indicated that her mother had prepared the preserves during the period 1950–1953, but, because she was a young child at the time, she was unable to recall the precise method(s) by which the preserves were prepared. Other than intermittent storage, the musty-smelling basement was largely unused, and a heavy layer of dust had accumulated on the preserves (Supplemental Video V2). Preserves were chosen for analysis if they showed no evidence of desiccation or microbial growth inside the jar and, secondarily, had a label with a date. Based on these criteria, a total of 31 sealed glass jars of preserved foods were identified and returned to the lab for SPME analysis. Following photo documentation of the preserves (Figure S2), all preserves were stored at room temperature (~19–21 °C) in plastic totes to shield them from light. After SPME sampling, preserves were stored in a refrigerator at 4 °C until the qualitative analysis described below was conducted.

Preserves were tentatively identified initially by visual examination in consultation with the family that donated them and staff members of the Moody County Museum, who possess considerable expertise in the foods traditionally canned in the region during this period. The identities of some preserves (e.g., pickled dill and sweet cucumbers, tomatoes, and apples) were confirmed via visual inspection relatively easily; however, roughly half of the preserves required further identification by smell and taste after SPME analysis was completed. A compiled tasting video of representative tests is presented in Supplemental Materials (Supplemental Video V1; Table S4). Unique and replicate sample types were established via smell and taste assessment in consultation with family members and Moody County Museum staff. Nearly all of the preserves were confirmed to a reasonable degree of certainty after smell and taste tests, but those we have identified as “head cheese,” “apricots,” and “crab apples” remain tentative (Table 1, Figure S2). 

### 3.3. SPME Analysis 

Prior to SPME sampling, the SPME fiber was conditioned according to the manufacturer’s instructions, and all glass jars were wiped with a large Kimwipe soaked with 95% ethanol to remove dust and external contaminants. The ethanol was allowed to evaporate in a fume hood for 15 min prior to SPME sampling to avoid it being absorbed onto the SPME fiber. Cleaned jars of preserve were placed in the middle of a 1.75 ft^3^ (~50 L) AtmosBag directly adjacent to a 2 L beaker with an adjustable lab clamp affixed to it for holding the SPME during sampling (Figure S1). A beaker was used in this instance because ring stands proved too tall and tended to damage the bag during manipulation of the jars for SPME sampling within the evacuated bag. The sampling bag was sealed and clamped shut after sample introduction according to manufacturer recommendations, and the entrained air was pumped out through a gastight vacuum hose connected to the bag. To ensure that the SPME did not absorb atmospheric contaminants, and to create a small volume (~1 L) inside the bag to permit easier manipulation of the sample and the SPME apparatus, the bag was inflated with ~1 L of argon gas. Once the bag was inflated, the lid was removed from the sample and the SPME fiber was deployed into the mouth of the jar about 2 centimeters above the sample surface (Figure S1). After 120 min, the SPME fiber was retracted, the SPME removed from the bag, and the SPME was immediately inserted into the GC-MS injector port for analysis. During the GC-MS run that followed (30 min), the sampling bag was purged with high velocity lab air to remove any residual moisture and/or volatile residues before the next SPME sampling period. Once the GC-MS run was complete, the same sample was again sealed in the bag and resampled with SPME as described above. This was repeated three times for each sample, with the aim of assessing volatile intensity and functional class composition with time. After three successive SPME samplings (6 h total sampling time), and prior to tasting, the pH of the preserves was determined (Table 1). 

The SPME sampling method was first developed and validated using store-bought savory and sweet preserves (Vlasic dill pickle spears and ShurFine maraschino cherries), to encompass a suite of representative compounds and compound classes believed relevant to those likely to comprise the volatile inventories of the historical preserves. To assess method sensitivity and reproducibility, authentic BPA standards were prepared and spiked into store-bought pickles and cherries and sampled with SPME. Informed by preliminary spiking trials and determinations of incurred BPA concentrations in selected preserves obtained from the basement and sacrificed for this purpose, and reviews of the relevant literature, 1000 μg of BPA (solubilized in ethanol) was deemed optimal and was spiked directly into the pickles/cherries. BPA-spiked preserves were then sealed inside the sampling bag as described above and sampled with SPME at 30, 60, 120, 240, and 360 min in triplicate to assess time to equilibrium uptake. Each of the triplicate samples was derived from SPME sampling of a freshly opened jar of each preserve to more accurately represent the sampling conditions under which the historical preserves were opened for the first time at the time of sampling. Equilibrium uptake, as measured by maximum total integrated chromatographic peak area, was reached by 120 min for both pickles and cherries (Figure 1A). This sampling time was used for SPME analysis of all the historical preserves as described above. The between- and within-sample precision of the method was assessed by SPME sampling of BPA-spiked pickles (N = 5) and cherries (N = 5), and then by three successive SPME samplings of three jars of each these same preserves (Figure 1B). 

As there have been no reports of the volatile inventory of Vlasic dill pickles against which we could compare our SPME-detected volatile inventory, we sought to further assess the efficacy of the method for this product by using it to profile volatiles emitted from the separate components of a pickled cucumber mixture. To achieve this, store-bought cucumbers were cut into spears of the same dimensions as the Vlasic dill pickle spears (sold in the 16 ounce jar), placed into washed and solvent-rinsed Vlasic jars, and then sampled via SPME as described above. To ensure there was no compound carryover from the original Vlasic pickle mixture, the recycled Vlasic jars were first sampled with SPME while empty. The remainder of the cucumber spears were placed into cleaned Vlasic jars (N = 5), which were filled to the neck with a lab-prepared approximation of the Vlasic dill brine. To ensure the lab-prepared dill brine was as close in composition to the Vlasic brine as possible, the concentration of vinegar (acetic acid) in the latter was determined via titration with sodium hydroxide (first standardized via potassium hydrogen phthalate (KHP)) and found to be 0.915 ± 0.0353 % *v*/*v*. The lab-prepared brine was then prepared by adding 0.184 L white vinegar to 0.816 L of distilled water into which 4.220 g and 0.465 g of dill seeds and table salt, respectively, were added. The lab brine was stirred aggressively with a magnetic stir bar set close to its maximum setting for 90 min. The lab-prepared brine solution was then used to pickle the fresh cucumber spears at room temperature (~19–21 °C) for 7 days prior to SPME analysis. The lab-prepared brine and the pickled cucumber spears were then each individually sampled with the SPME method in triplicate, as described above, to define their volatile profiles (Table S2).

### 3.4. SPME QA/QC

To ensure no cross contamination between samples and sampling replicates, the sampling bags were purged with high velocity lab air for 30 min before and after each jar was sampled with SPME (and between replicate samplings of the same jar). Each sampling bag containing only argon gas, and the SPME apparatus was sampled with SPME for 120 min after every five jars analyzed to serve as a procedural blank. To ensure bag integrity, sample bags were discarded after each set of seven preserves analyzed. Butylhydroxytoluene (*m/z* 220) was consistently detected in the procedural blanks, determined as direct off gassing from the bag and from the adhesive used to affix the lab clamp (holding the SPME sampler) to the beaker. This compound was excluded from the analysis. To ensure the SPME fiber was free of contaminants prior to each SPME sampling period, the fiber was heated at 250 °C in the GC injector for 30 min and a TIC was generated to track contaminant desorption from the fiber. SPME fibers never required more than a single heating period in the GC injector to remove all contaminants accumulated while stored between sampling periods.

### 3.5. GC-MS Analysis

VOCs were desorbed from the SPME fiber via direct insertion into the heated injector port of a QP2010 SE GC-MS (Shimadzu, Kyoto, Japan), equipped with a SPME injector liner (0.75 mm; Restek Corporation, Bellefonte, PA, USA) and Rxi-5ms capillary column (30 m x 0.25 mm with a 0.25 μm 5% diphenyl 95% dimethyl polysiloxane phase; Restek). Helium was used as the carrier gas. The SPME fiber was desorbed for 30 min. GC injector temperature was maintained at 250 °C and operated in the splitless mode with a helium flow rate of 1.15 mL min^−1^ through the column (8.4 mL min^−1^ total flow). The initial GC column temperature was set to 40 °C, ramped to 100 °C at 5 °C min^−1^ with a 3 min hold, and then ramped to 250 °C at 10 °C min^−1^. The MS was operated in the electron impact ionization mode at 70 eV and 0.1 kV detector voltage. The ion source and MS interface temperatures were maintained at 260 °C and 270 °C, respectively. Mass spectra were obtained in the full scan mode, with the mass range 30–500 amu, scanned at a rate of 2000 scans/s. 

### 3.6. Data Analysis

TICs of preserve samples and procedural blanks were initially overlaid for preliminary comparison and identification of compounds occurring in both. Compounds present in both the samples and blanks were excluded from analysis. TIC and mass spectral analyses were performed using the Shimadzu GCMSsolution software (Version 4.11; Shimadzu, Kyoto, Japan). Individual compounds were identified using authentic standards and Kovats retention indices in conjunction with comparisons of measured mass spectra with reference spectra contained in the NIST/EPA/NIH Mass Spectral Library (NIST 14, Version 2.2, Gaithersburg, MD, USA). Only TIC peaks with S/N > 3 and mass spectral library similarity index > 80% were considered positively identified compounds. All other TIC peaks were excluded from analysis, which necessitated an estimated 40% of detected compounds being excluded from analysis. A unique compound was defined as the singular detection of that compound in each sample conforming to these detection criteria. Percentage contributions of sampled compound classes and unique compounds to total integrated TIC peak areas were computed using only the sum of all peak areas conforming to these detection criteria. Spiked and naturally incurred BPA concentrations were quantified using as 6 point calibration curve prepared from serial dilutions of authentic standards (Figure S5). All data reduction and statistical analysis was performed with Excel (Microsoft, Redmond, WA, USA), StatPlus (AnalystSoft, Walnut, CA, USA), and Prism (GraphPad, San Diego, CA, USA) software. 

## 4. Conclusions

The results presented here demonstrate that SPME sampling is a viable and reproducible method for volatile profiling of rare historical preserved foods. Additionally, the use of an AtmosBag as a chamber that can be sealed, vacuumed, and purged presents a relatively simple and inexpensive means of assessing volatile emissions from any large and/or oddly-shaped container. As the method was developed with modern store-bought preserved foods and was applied to historical preserves, it likely represents a generally accessible means of sampling the volatile inventory of any food type stored in a container. The jars assessed in this work opened with screw-top lids, so additional modifications may be necessary if foods sealed by other means were to be assessed using this method. This work represents one of the first efforts to profile the volatile inventories of rare historical preserved foods, and presents one of the oldest instances of BPA detection in foodstuffs. 

## Figures and Tables

**Table 1:** Quantitative and qualitative data for the preserved foods assessed in this study.

Sample ^a^	Sample Number	N ^b^	pH	Sample Description ^c^	Taste ^d^
Apple 1	1, 15	2	3.1–3.4	Sliced and shredded apple in opaque liquid with apple sauce consistency	Sweet
Apple 2	8	1	3.0	Sliced apples stacked in clear liquor	Sweet
Apricots	17, 27, 28	3	2.9–3.5	Spherical, fleshy fruit bodies in dark liquid	Sweet
Brandied Fruit	4, 23	2	2.9–3.3	Dark gelatinous fruit bodies resembling plums in dark, viscous liquor	Sweet/alcohol
Crab Apple 1	25	1	3.4	Round fleshy fruit bodies with a single stem and many small seeds in the center in dark liquid	Sweet
Crab Apple 2	26	1	3.8	Round fleshy fruit bodies with no stem and many small seeds in the center in dark liquid	Sweet
Dill Pickle 1	19	1	3.8	Whole cucumbers with dill plants and seeds in clear liquid—smaller jar	Salty
Dill Pickle 2	29, 30	2	3.2–3.3	Cucumbers quartered longitudinally with dill plants and seeds in clear liquid—larger jar	Salty
Head Cheese	5	1	3.7	Cylindrical mass of spongy material resembling cheese in amber liquor	Salty/savory
Mincemeat 1	3, 12	2	3.9–4.0	Fleshy fruit bodies resembling currants, raisins, leafage, and fleshy chunks resembling meat in small volume of clear liquid—larger jar	Sweet/savory
Mincemeat 2	13	1	4.0	Fleshy fruit bodies resembling currants, raisins, leafage, and fleshy chunks resembling meat in small volume of clear liquid—smaller jar	Sweet/savory
Peaches	7	1	3.6	Dark, spherical, fleshy fruit bodies with a single solid pit in the center in dark liquid	Sweet
Rhubarb 1	9, 11, 21, 22	4	2.9–3.0	Shredded vegetative material in dark liquid	Slightly sweet
Rhubarb 2	24	1	3.1	Cylindrical mass of vegetative material in dark liquid	Slightly sweet
Sweet Pickle 1	6, 18	2	2.5–3.2	Halved cucumbers in dark liquid	Sweet
Sweet Pickle 2	20	1	3.2	Cucumber slices in dark liquid—smaller jar	Sweet
Sweet Pickle 3	31	1	3.2	Cucumber slices in dark liquid—larger jar	Sweet
Tomato 1	2, 10, 16	3	4.1–4.2	Whole and shredded tomato in opaque, red liquor with tomato soup consistency	Sweet/salty
Tomato 2	14	1	4.1	Whole and shredded tomato in red liquor with spaghetti sauce consistency	Sweet/salty

^a^ Sample identification and determination of unique sample types and replicates of the same sample type was achieved via combination of anecdotal accounts of method/time of preparation by the donating family and our own examinations of appearance, smell, and taste. ^b^ Represents the number of replicates of the same sample type. ^c^ Sample descriptions are derived from visual inspections of the samples by the authors to arrive at consensus characterizations. ^d^ Taste descriptions are derived from tastings performed by author MOG (Supplemental Video V1).

## Supplementary Materials

The following are available online: Schematic of SPME sampling in the AtmosBag (Figure S1); photodocumentation of the 31 historical preserves sampled in this work (Figure S2); analysis of the total integrated area and the breakdown of compound classes for each of the historical preserves assessed (Figure S3); amount of BPA per sample (Figure S4); standard curve to quantify BPA (Figure S5); raw data of compound classes used to create Figure 3 (Table S1); compounds listed are those detected during three independent 2 h SPME samplings and aggregated to display the complexity of the volatile profile (Table S2); the masses of the historical preserves (Table S3); sample numbers and video start times for each of the historical preserves taste tested in Supplementary Video V1 (Table S4); all data collected in this study (Supplementary Data File 1); visual documentation of taste tests conducted by author M.O.G. on representative historical preserve samples (Supplementary Video V1); video documentation of the basement where the historical preserves were stored since the period 1950–1953 (Supplementary Video V2).
