# Supplementary material for: Profiling Volatile Constituents of Homemade Preserved Foods Prepared in Early 1950s South Dakota (USA) Using Solid-Phase Microextraction (SPME) with Gas Chromatography–Mass Spectrometry (GC-MS) Determination
Source: Molecules. 2019 Feb 13;24(4):660. doi: 10.3390/molecules24040660 (PMC6413207; doi:10.3390/molecules24040660)
Supplement: Supplementary file 1 [file molecules-24-00660-s001.zip › Supplementary Material.pdf]

Profiling Volatile Constituents of Homemade Preserved Foods Prepared in Early 1950s South Dakota (USA) Using Solid-Phase Microextraction (SPME) with Gas Chromatography-Mass Spectrometry (GC-MS) Determination

Lucas J. Leinen[a,§], Vaille A. Swenson[b,§], Hope L. Juntunen[a,b], Scott E. McKay[a], Samantha M. O'Hanlon[c], Patrick Videau[d,*], Michael O. Gaylor[a,*]

[a]Department of Chemistry, Dakota State University, Madison, SD 57042, USA

[b]Department of Biology, Dakota State University, Madison, SD 57042, USA

[c]School of Psychological Science, Oregon State University, Corvallis, OR, 97331, USA

[d]Department of Biology, Southern Oregon University, Ashland, OR, 97520, USA

§ Both authors contributed equally to the work.

*Corresponding authors.

*Michael O. Gaylor
Department of Chemistry, Dakota State University, 820 Washington Avenue N, Madison, SD 57042, USA
Office Number: 001 605 256 5822
Fax Number: 001 605 256 5643
E-mail address: michael.gaylor@dsu.edu

*Patrick Videau
Department of Biology, Southern Oregon University, 1250 Siskiyou Boulevard, Ashland, OR, 97520, USA
Office Number: 001 541 552 6788
Fax Number: 001 541 552 7127
E-mail address: videaup@sou.edu

Supplementary Material

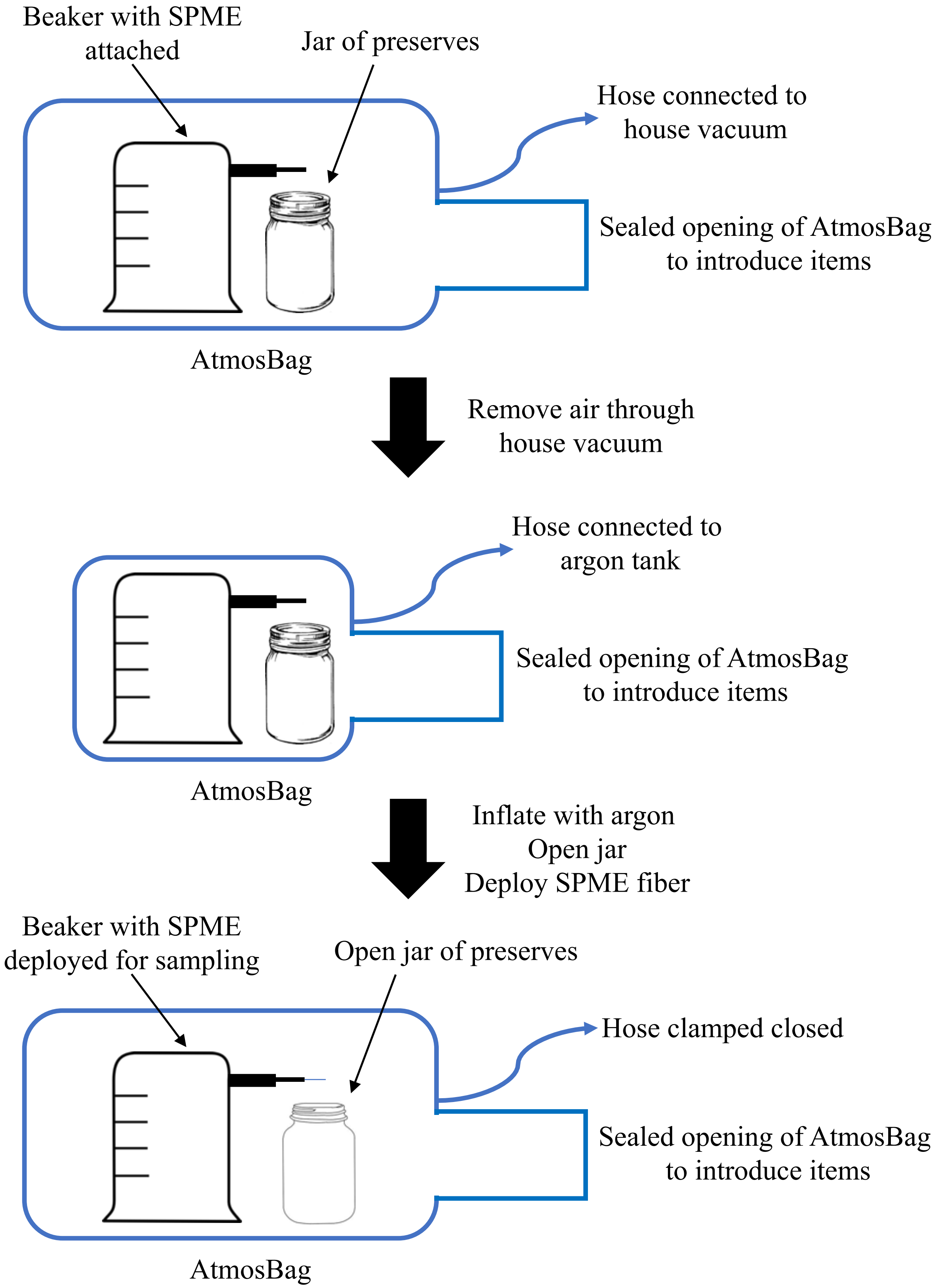

Figure S1. Schematic of the SPME sampling protocol used in this work.

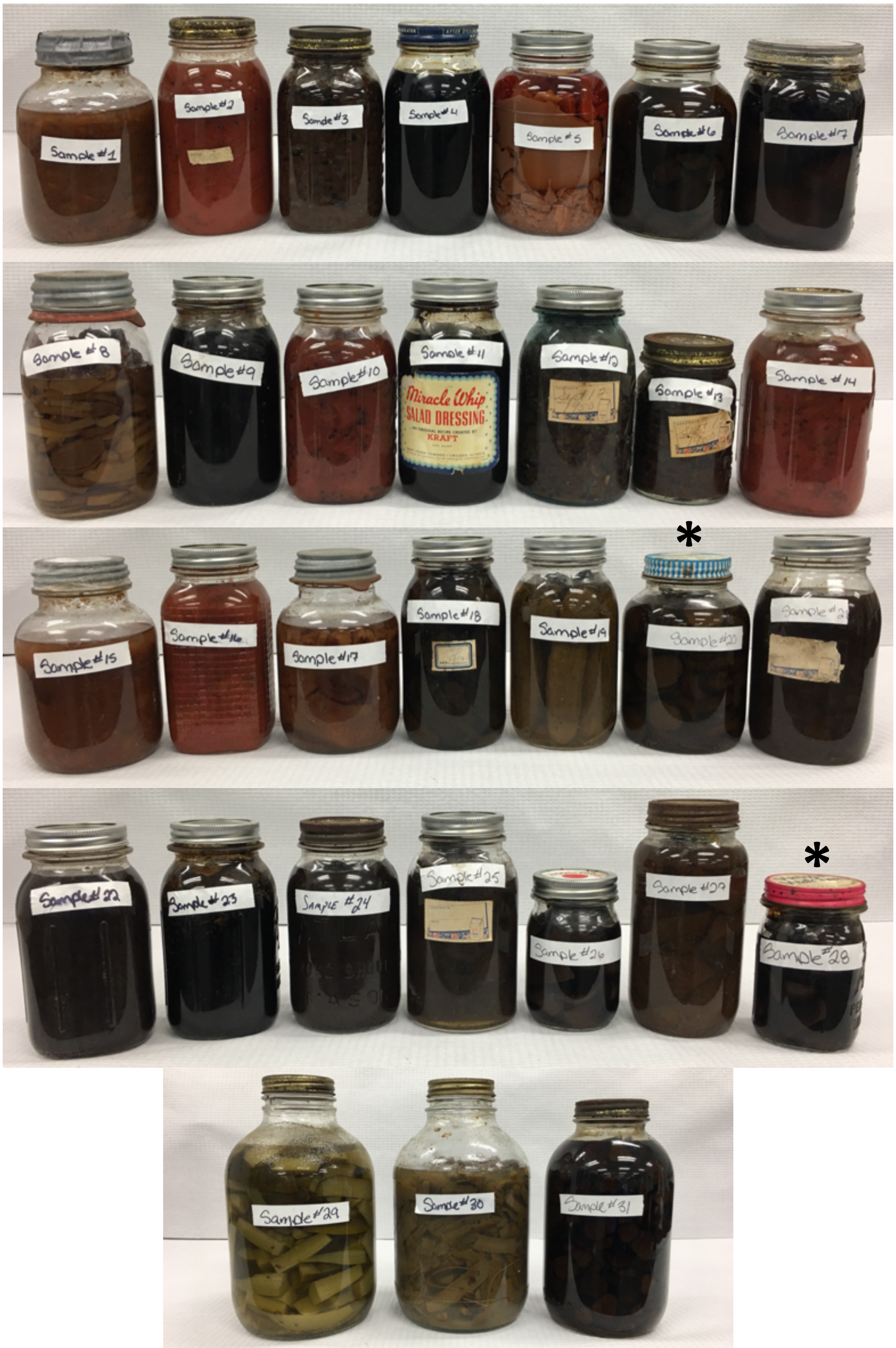

Figure S2. Photodocumentation of the 1950s preserved foods analyzed in this study after they were wiped down with 95% ethanol to remove dust and debris. Jars are aligned sequentially and the white label displays the sample number. Identifying markings on the jars are also included in the photographs for accuracy. An asterisk is included above jars that did not yield BPA above the limit of detection.

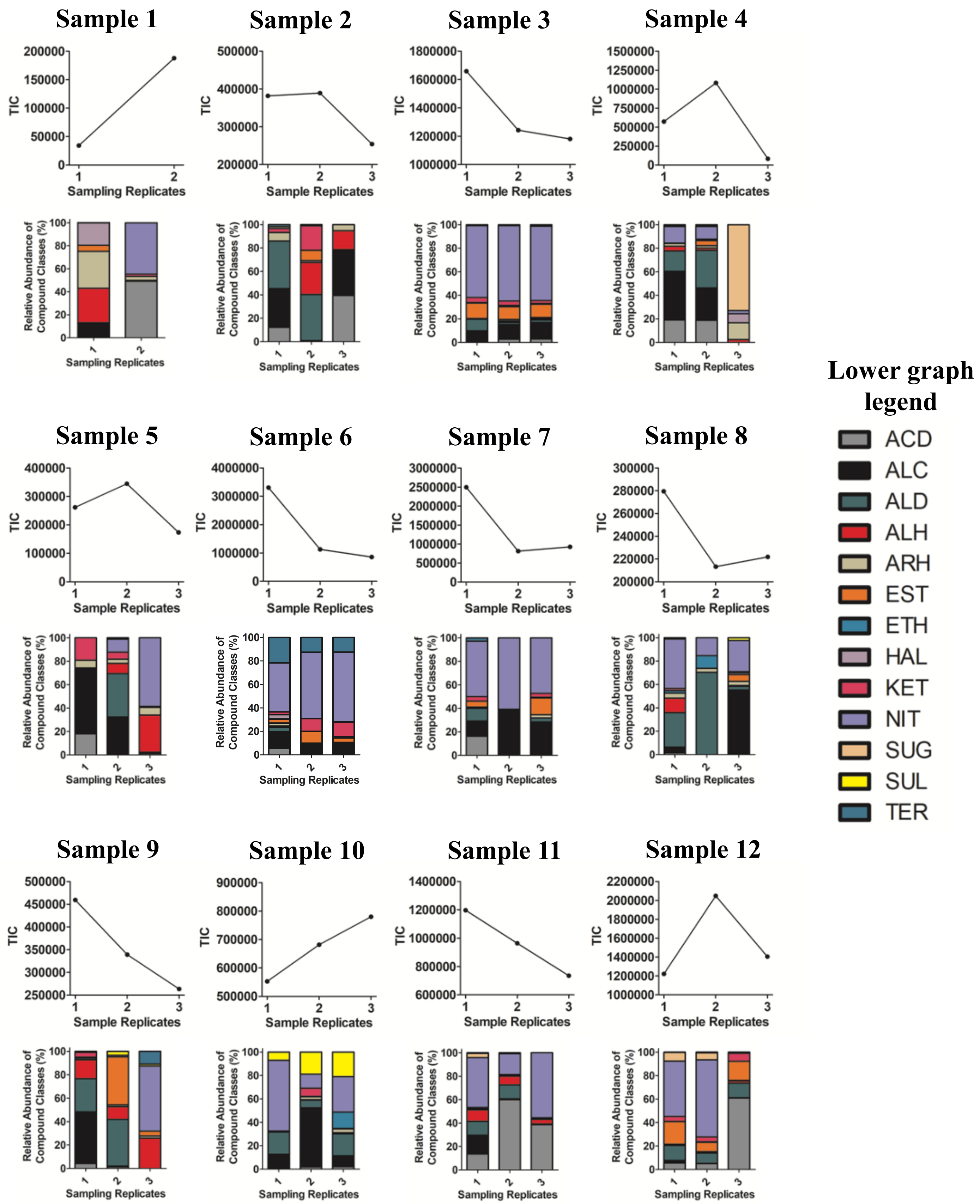

Sample 1
Sample 2
Sample 3
Sample 4
Sample 5
Sample 6
Sample 7
Sample 8
Sample 9
Sample 10
Sample 11
Sample 12
TIC
Sampling Replicates
Sample Replicates
Relative Abundance of Compound Classes (%)
Lower graph legend
ACD
ALC
ALD
ALH
ARH
EST
ETH
HAL
KET
NIT
SUG
SUL
TER

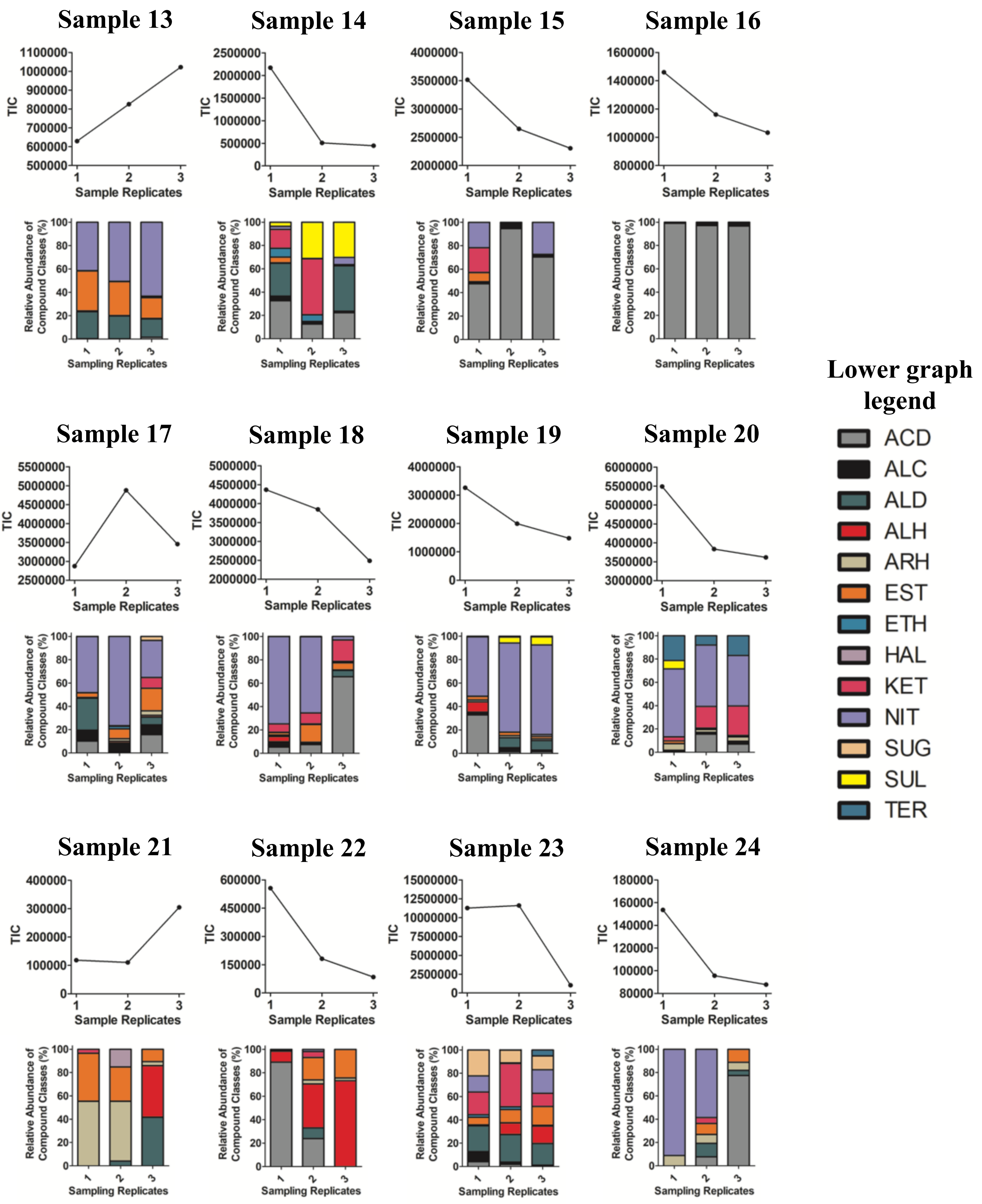
Sample 13
Sample 14
Sample 15
Sample 16
Sample 17
Sample 18
Sample 19
Sample 20
Sample 21
Sample 22
Sample 23
Sample 24
TIC
Sample Replicates
Relative Abundance of Compound Classes (%)
Sampling Replicates
Lower graph legend
ACD
ALC
ALD
ALH
ARH
EST
ETH
HAL
KET
NIT
SUG
SUL
TER

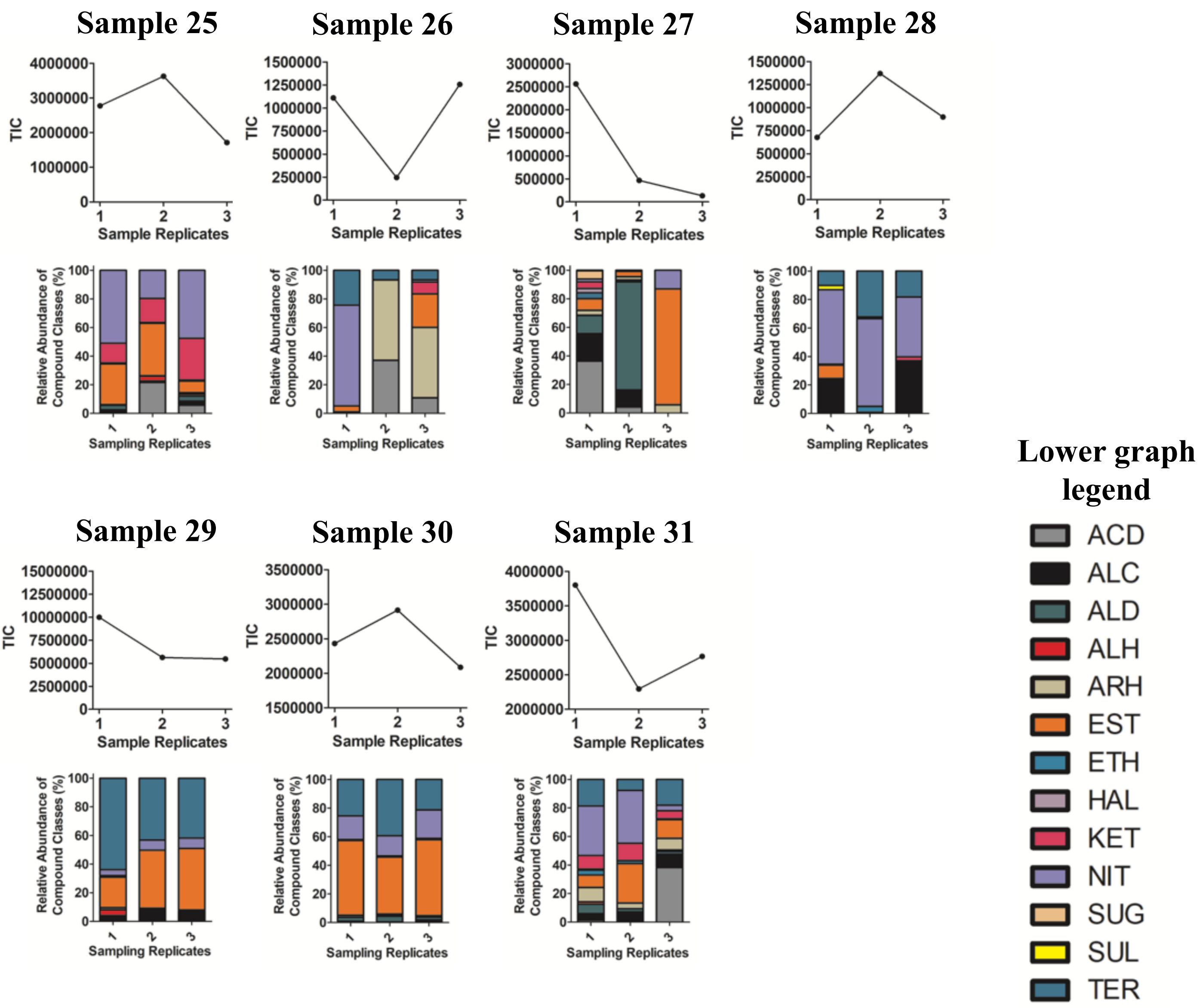

Figure S3. Assessment of the volatile profile of 31 historical preserve samples. The data from each of the three successive samplings of each historical preserve are presented as the total integrated area from the chromatograms of compounds with a NIST score over 80% (top graph), and the relative abundance of the compounds belonging to each compound class assessed here (bottom graph). The legend for the column colors used in the bottom graph for each sample is on the right side of the page. Compound classes are defined as follows: ACD = acids; ALC = alcohols; ALD = aldehydes; ALH = aliphatic hydrocarbons; ARH = aromatic hydrocarbons; EST = esters; ETH = ethers; HAL = halogen-containing; KET = ketones; NIT = nitrogen-containing; SUG = sugar alcohols; SUL = sulfur-containing; TER = terpenoids.

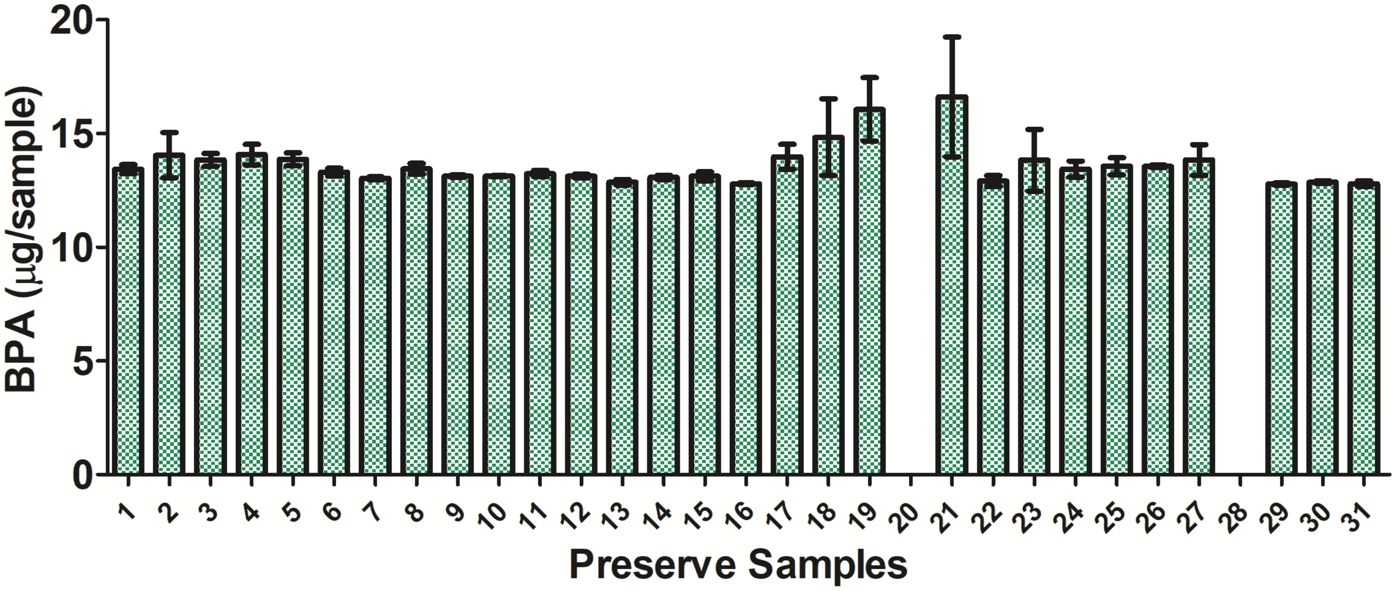

Figure S4. The concentration of BPA in historical preserves is presented as the average ± standard deviation normalized to absolute mass detected per sample as measured from three successive samplings of each historical preserve.

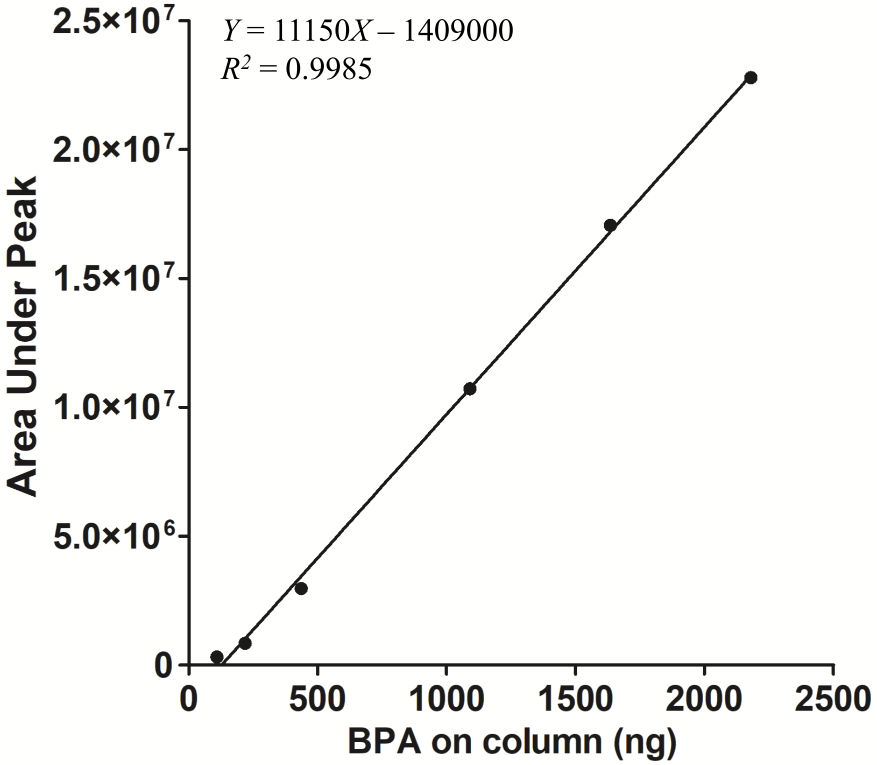

Figure S5. Quantitation curve created with the an authentic BPA standard generated over the concentration range of 100 to 2200 ng on-column in triplicate.

Table S1. Quantification of the relative abundance of each compound class by sample used to create Figure 3. For multiple preserve types, the preserve name is followed by the sample number in parentheses, e.g. Apple 1 (1). The numbers in each compound class are the percentage of this compound class detected in the first (2-hour) SPME sampling of each jar. Percentages were computed by summing the total integrated peak areas for each of the compounds of a given class and dividing it by the total integrated peak area in the TIC. The absence of compounds in a given class is presented as an empty box.

**Sample**	**ACD**	**ALC**	**ALD**	**ALH**	**ARH**	**EST**	**ETH**	**HAL**	**KET**	**NIT**	**SUG**	**SUL**	**TER**
Apple 1 (1)		13.23		29.87	32.13	5.16		19.60					
Apple 1 (15)	47.58	1.29	0.15	0.16	0.22	7.69	0.12		21.08	21.70			
Apple 2 (8)	1.54	4.88	29.49	12.43	4.30		2.29		1.73	42.32		1.02	
Apricots (17)	10.13	9.39	27.53	0.10	0.44	4.01			0.16	48.09		0.13	
Apricots (27)	36.39	19.10	12.84	0.15	3.46	8.17	4.18	2.99	4.64	1.91	5.87	0.29	
Apricots (28)		24.64				9.32			0.75	52.29		2.92	10.08
Brandied Fruit (4)	19.13	41.26	17.28	4.01	2.52				0.20	14.04		0.67	0.89
Brandied Fruit (23)	4.13	8.66	22.18	0.49	0.26	6.48	2.31		19.46	13.86	22.18		
Crab Apple 1 (25)		2.52	3.27		0.27	28.58	0.60		13.92	50.84			
Crab Apple 2 (26)		0.17			0.97	4.10			0.04	70.43			24.28
Dill Pickle 1 (19)	32.84	2.16	0.16	8.85	1.66	3.08			0.07	50.58			0.61
Dill Pickle 2 (29)		4.03		3.94	1.70	21.28	0.57	0.19	0.29	4.13			63.88
Dill Pickle 2 (30)	0.42	0.34	2.99		1.34	52.28	0.32		0.31	16.54			25.46
Head Cheese (5)	18.08	56.17			6.61				19.13				
Mincemeat 1 (3)	0.96	8.91	9.85		0.66	13.00	0.10	0.41	4.24	61.05		0.55	0.27
Mincemeat 1 (12)	5.61	2.14	12.74	0.44	0.54	19.27	0.14		4.44	47.02	7.42	0.24	
Mincemeat 2 (13)	0.44		22.99		0.61	34.24			0.24	41.48			
Peaches (7)	16.36	12.88	10.91	0.67	0.21	5.19			3.78	47.18			2.82
Rhubarb 1 (9)	4.29	44.02	28.38	16.19	1.32			0.77	4.29			0.75	
Rhubarb 1 (11)	13.64	15.96	11.83	9.81	0.66	0.35		1.08		42.54	3.57	0.55	
Rhubarb 1 (21)					55.37	41.24			3.38				
Rhubarb 1 (22)	89.08			9.42	0.20	1.30							
Rhubarb 2 (24)	7.69		11.63		7.55	9.43			5.07	58.64			
Sweet Pickle 1 (6)	0.27	8.53		0.69	1.02	3.90	1.06		12.48	59.62			12.44
Sweet Pickle 1 (18)	5.29	3.45	1.16	4.58	1.30	1.95	0.34		7.17	74.71		0.05	
Sweet Pickle 2 (20)	0.32	0.32	0.74	0.44	5.63	2.26			3.56	58.32		7.13	21.29
Sweet Pickle 3 (31)	1.41	4.53	6.63	1.62	10.14	8.69	3.48	0.62	9.52	34.70		0.07	18.59
Tomato 1 (2)	12.32	33.04	40.58		7.30				3.48			1.56	1.72
Tomato 1 (10)		12.65	18.95		1.00					60.37		7.03	
Tomato 1 (16)	99.05				0.15	0.16			0.10	0.38	0.16		
Tomato 2 (14)	32.61	4.04	28.14	0.15	0.28	4.86	7.57		16.15	2.64		3.58	

Compound classes are defined as follows: ACD = acids; ALC = alcohols; ALD = aldehydes; ALH = aliphatic hydrocarbons; ARH = aromatic hydrocarbons; EST = esters; ETH = ethers; HAL = halogen-containing; KET = ketones; NIT = nitrogen-containing; SUG = sugar alcohols; SUL = sulfur-containing; TER = terpenoids.

Table S2. Comparison of the compounds tentatively identified during SPME analysis of Vlasic dill pickles, cucumbers, lab-made brine, and cucumber spears steeped in the lab-made brine. The compounds listed are those derived from three independent 2 h SPME samplings and aggregated to display the complexity of the volatile profile.

RT (min)	Compounds[a]	RI[b]	Vlasic Dill Pickles	Lab Prepared Constituents[c]		
				Cucumber[d]	Brine	Cucumber + Brine
1.8	Acetic acid	600	x	-	x	x
1.9	Ammonium acetate	-	x	-	x	x
2.0	Ethyl acetate	628	x	-	x	x
5.5	Diallyl sulfide	-	x	-	-	-
8.9	β-Myrcene	992	x	-	x	x
9.2	1-(2-methoxy-1-methylethoxy)-2-propanol	999	x	-	-	-
9.3	2-(2-ethoxyethoxy)-ethanol	-	x	-	-	-
9.4	α-Phellandrene	1006	x	-	x	x
9.6	1-(2-methoxypropoxy)-2-propanol	-	x	-	-	-
9.7	(+)-4-Carene	1009	x	-	x	x
9.9	p-Cymene	1027	x	-	x	x
10.0	Limonene[c]	1030	x	x	-	x
12.8	cis-1-methyl-4-(1-methylethyl)-2-cyclohexen-1-ol	1145	x	-	-	-
15.2	3,6-dimethyl-2,3,3a,4,5,7a-hexahydrobenzofuran	1187	x	x	-	x
15.5	α-Terpineol	1195	x	-	-	-
15.7	trans-2-methyl-5-(1-methylethenyl)-cyclohexanone	1200	x	-	x	x
17.2	D-Carvone	1253	x	-	x	x
17.3	(S)-2-methyl-5-(1-methylethyl)-2-cyclohexen-1-one	1256	x	-	-	-
17.9	Nonanoic acid	1275	x	-	-	-
21.8	1-Dodecanol	1577	x	-	-	-
	**Total Compounds**		**20**	**2**	**9**	**11**

"x" denotes compound detected.
"-" denotes compound not detected.
[a]All compounds identified at ≥80% similarity to the NIST Mass Spectral Library.
[b]Kovats retention index
[c]Artificial brine was prepared with as many ingredients as listed on the nutritional labeling of the Vlasic pickles. Cucumbers were purchased from a local supermarket, cut into spears of the same dimensions as the Vlasic pickles, and packed into washed and solvent-rinsed Vlasic pickle jars.

[d]Limonene is presumed to be an artifact of chitosan coatings commonly used to extend cucumber shelf life, or perhaps the result of a metabolic stress response to preparation (Bruni et al. 2007). Bruni, R.; Bianchi, A.; Bellardi, M.G. Essential Oil Composition of Agastache anethiodora Britton (Lamiaceae) Infected by Cucumber Mosaic Virus (CMV). Flavour and Fragrance Journal 2007, 22, 66-70.

Table S3. Masses of the historical preserves were determined by weighing the jars prior to sampling but after they were wiped with 95% ethanol and allowed to dry in a fume hood.

Sample	Mass (g)
1	1319.9
2	1244.8
3	1400.1
4	1426.8
5	1382.0
6	1441.6
7	1466.2
8	1179.1
9	1471.6
10	1249.0
11	1393.3
12	1460.4
13	797.3
14	1259.3
15	1320.7
16	1269.1
17	1350.2
18	1388.2
19	1250.8
20	1421.7
21	1319.9
22	1385.8
23	1564.3
24	1387.5
25	1491.6
26	704.9
27	1305.3
28	716.8
29	3794.3
30	3490.9
31	3007.9

Table S4. Start times of the video documentation of the qualitative taste tests of representative historical preserve samples.

**Sample**	**Start time in Video V1**
1	0:01
2	0:39
3	1:22
4	2:39
5	3:46
6	4:58
7	5:55
11	7:09
19	8:20
20	9:09
25	10:00
26	11:30
29	12:26
31	13:15
